# Optimizing biogas production from poultry manure and cocoa pod husks co-digestion: implications for circular bioeconomy and decentralized energy systems in West Africa

**DOI:** 10.3389/fbioe.2026.1845247

**Published:** 2026-06-17

**Authors:** Ako Pierre Elischama Brou, Djangbadjoa Gbiete, Yawovi Nougbléga, Satyanarayana Narra

**Affiliations:** 1 West African Science Service Center on Climate Change and Adapted Land Use (WASCAL), University of Lomé, Lomé, Togo; 2 Material and Energy Valorisation of Biogenous Residues, Waste and Resource Management, Faculty for Agriculture, Civil and Environmental Engineering, University of Rostock, Rostock, Germany; 3 Centre d’Excellence Régional pour la Maîtrise de l’Électricité (CERME), Université de Lomé, Lomé, Togo

**Keywords:** anaerobic co-digestion, biogas optimization, carbon-to-nitrogen ratio, circular bioeconomy, cocoa pod husk, mesophilic and thermophilic digestion, poultry manure

## Abstract

**Introduction:**

Rapid population growth and agro-industrial expansion in West Africa are intensifying organic waste accumulation and energy insecurity, necessitating scalable waste-to-energy strategies.

**Methods:**

Batch biogas potential assessment assays were conducted using the ANKOM system under mesophilic (37°C) and thermophilic (55°C) conditions at C/N ratios of 20, 25, 30, and 35 using poultry manure (PM) and cocoa pod husk (CPH).

**Results:**

Under mesophilic mono-digestion, PM and CPH yielded 210.73 ± 27.12 and 244.65 ± 8.48 mL/g VS, respectively. Co-digestion significantly improved performance, with maximum cumulative biogas production of 348.65 ± 10.44 mL/g VS at C/N 25 under mesophilic conditions, corresponding to increases of approximately 65% and 42% compared with PM and CPH mono-digestion, respectively. Statistical analysis confirmed a significant effect of C/N ratio (p < 0.001) and a significant interaction between C/N ratio and temperature (p = 0.031).

**Discussion:**

Maintaining a balanced C/N ratio (25–30) under mesophilic conditions provides a technically robust and energy-efficient pathway for decentralized biogas systems. These findings support low-input anaerobic digestion strategies for circular bioeconomy development and sustainable energy access in West Africa.

## 1 Introduction

Rapid population growth, urbanization, and agricultural intensification across West Africa are increasing pressure on both waste management systems and energy supply infrastructures (Agbossou et al., 2022; Boakye-Yiadom et al., 2024). In many countries of the region, including Togo, energy demand remains largely dependent on traditional biomass and imported fossil fuels, while agricultural residue management is often poorly organized. As a result, large quantities of organic wastes are openly dumped or left to decompose uncontrolled, contributing to greenhouse gas (GHG) emissions, environmental pollution, nutrient losses, and sanitation challenges (Kiss et al., 2023; Preuss and You, 2023). In Togo alone, national GHG emissions were estimated at approximately 21 million tons CO2-equivalent in 2018, with agriculture and energy identified among the dominant contributing sectors (Agbossou et al., 2022). These challenges have intensified interest in decentralized waste-to-energy pathways capable of simultaneously supporting renewable energy generation, residue valorization, and circular bioeconomy development, particularly through the recovery of value from underutilized agricultural biomass streams and organic residues (Ayua et al., 2026).

Among the major agricultural residues generated in Togo, poultry manure (PM) and cocoa pod husk (CPH) represent abundant yet insufficiently valorized organic resources. Poultry production generates large volumes of nitrogen-rich manure whose uncontrolled accumulation can promote ammonia volatilization, odor emissions, pathogen dissemination, and nutrient leaching into surrounding ecosystems (Kabelitz et al., 2021; Kiss et al., 2023). Similarly, cocoa processing produces substantial quantities of lignocellulosic pod husks that are frequently discarded, openly burned, or left to decay in fields, thereby contributing to particulate emissions and phytopathogenic proliferation, including fungal infections such as *Phytophthora palmivora* (Mboua et al., 2020; Ouattara et al., 2021). Despite these environmental burdens, both residues possess considerable organic and energetic potential and therefore constitute promising feedstocks for anaerobic digestion (AD) systems.

AD is widely recognized as an effective biological conversion pathway for transforming organic wastes into biogas and nutrient-rich digestate while simultaneously mitigating uncontrolled methane emissions (Francisco López et al., 2024; Gadirli et al., 2023). Beyond renewable energy generation, AD contributes to nutrient recycling, decentralized energy access, and climate-smart agricultural practices. However, process stability and biogas productivity remain strongly dependent on substrate characteristics and operational parameters, particularly the carbon-to-nitrogen (C/N) ratio and digestion temperature (Bardi and Oliaee, 2021; Lee et al., 2024). Low C/N substrates such as PM may induce excessive ammonia accumulation and methanogenic inhibition, whereas highly lignocellulosic residues such as CPH may exhibit nitrogen limitation and slow hydrolysis kinetics (Suhartini et al., 2021). Consequently, maintaining balanced substrate stoichiometry is essential for sustaining microbial activity, buffering capacity, and stable biogas production. Previous studies generally identify an optimal C/N range between 20 and 30 for efficient anaerobic conversion (Choi et al., 2020).

To overcome the limitations associated with mono-digestion, co-digestion strategies combining carbon-rich and nitrogen-rich feedstocks have increasingly been proposed as a means of improving nutrient complementarity, microbial stability, and overall methane productivity (Atmowidjojo et al., 2023; Jasińska et al., 2023). Temperature regime further influences hydrolysis efficiency, microbial kinetics, and process robustness. Mesophilic digestion (35 °C–40 °C) generally offers greater operational stability and lower energy demand, whereas thermophilic digestion (around 55 °C) may accelerate organic matter degradation but often increases process sensitivity and ammonia-related inhibition risks (Hu and Shen, 2024; Wang et al., 2019). The interaction between substrate stoichiometry and temperature therefore represents a critical determinant of AD performance and operational reliability.

Despite increasing global interest in agricultural residue valorization, the anaerobic co-digestion of PM and CPH remains poorly characterized under West African production conditions. This limitation is scientifically important because AD performance is highly sensitive to substrate physicochemical variability, and optimization data generated from one geographical context may not be reliably transferable to another (Alengebawy et al., 2024; Trujillo-Reyes et al., 2024). In West Africa, PM composition may differ substantially from that reported in European or Asian systems due to variations in poultry feeding practices, bedding incorporation, climatic conditions, and manure storage methods, all of which influence nitrogen availability, ash content, and ammonia release during digestion (Ball et al., 2024; Tawfik et al., 2023). Likewise, the biochemical composition and biodegradability of CPH are affected by cocoa cultivar characteristics, tropical rainfall patterns, fruit maturity, and post-harvest management, which can modify lignin, cellulose, hemicellulose, and soluble phenolic fractions (Dewi et al., 2025; Martínez-Giraldo et al., 2026). Recent advances in renewable biomass valorization and circular bioeconomy systems further emphasize that feedstock complementarity and locally adapted substrate balancing are central to the design of efficient low-input bioenergy systems (Adnane et al., 2024; Koubaa, 2024). Nevertheless, experimental data evaluating the combined influence of C/N ratio and temperature regime on PM-CPH co-digestion under tropical West African conditions remain scarce. Accordingly, this study investigates the anaerobic co-digestion of locally sourced PM and CPH under varying C/N ratios (20, 25, 30, and 35) and mesophilic (37 °C) and thermophilic (55 °C) conditions using standardized biogas potential assessment assays. The study aims to identify the optimal substrate balance and temperature regime to maximize biogas yield and process stability, while providing context-specific data relevant to decentralized bioenergy deployment and agricultural residue valorization in West Africa.

## 2 Materials and methods

### 2.1 Substrate collection and pretreatment

PM was collected from the poultry farm of the École Supérieure d’Agronomie (University of Lomé, Togo). At this site, wood chips are routinely used as bedding material in poultry houses; consequently, the recovered poultry manure was naturally mixed with fine bedding residues, contributing to its fibrous structure and elevated inorganic fraction. Cocoa pod husks (CPH) were collected from cocoa plantations in Agou, located in southern Togo. Fresh samples of both substrates were transported to the laboratory in sealed plastic containers. The samples were dried at 105 °C to constant weight and ground. This drying and grinding step standardized moisture content and acted as a mild thermal–physical pretreatment that increased substrate surface area and partially disrupted cell walls, which could ultimately enhance initial hydrolysis rates during anaerobic digestion (Gbiete et al., 2024). Each substrate was stored in airtight polyethylene containers at room temperature until use.

The inoculum used for all biogas potential assessment tests was digestate collected from the Hofladen Hof Postma biogas plant in Lambrechtshagen, Germany. This plant operates a continuously stirred mesophilic anaerobic digester that treats a mixture of cow manure and straw as the primary feedstock. We note that regional differences in digester microbial communities (e.g., between German and West African systems) may affect initial biogas production rates and adaptation, so the batch tests here quantify the biogas potential of the substrate under a standard mesophilic inoculum rather than *in situ* performance in the Togolese context. After collection, the inoculum was transported to the laboratory and left to stabilize at ambient temperature for 1 week to allow degassing, during which residual, easily degradable compounds are consumed. This step ensured a biologically active yet stable inoculum, minimizing background gas production during assays.

### 2.2 Substrate physicochemical characterization

#### 2.2.1 Proximate analysis

PM and CPH were characterized in accordance with the DIN EN 15935 (2012-11) standard, following the procedure described by Gbiete et al. (2024), to determine their total solids (TS), moisture content (MC), ash content (AC), and volatile solids (VS).

#### 2.2.2 Fiber analysis

Fiber analysis was conducted to quantify the structural carbohydrate fractions of the substrates, namely, cellulose, hemicellulose, and lignin, using the Van Soest sequential fiber analysis method as described by Liebetrau and Pfeiffer (2020). This method allows for the successive determination of neutral detergent fiber (NDF), acid detergent fiber (ADF), and acid detergent lignin (ADL). Based on these fractions, the contents of cellulose, hemicellulose, and lignin (% of total solids, TS) were calculated using Equations 1–3, respectively.

Cellulose=ADF−ADL (1)

Hemicellulose=NDF−ADF (2)

Lignin=ADL (3)

#### 2.2.3 Ultimate analysis

Ultimate analysis was carried out by an external accredited laboratory to determine the elemental composition of the substrates, including carbon (C), hydrogen (H), nitrogen (N), sulphur (S), and oxygen (O), as well as their nutrient composition. The contents of C, H, and N were analyzed in accordance with DIN EN ISO 21663 (2021–03), while O and S were determined following DIN 51733 (2016–04) and DIN EN 15408 (2011-05), respectively. Based on these elemental data, the carbon-to-nitrogen (C/N) ratio was calculated. The concentrations of nutrients, namely, iron (Fe), calcium (Ca), potassium (K), magnesium (Mg), sodium (Na), and phosphorus (P), were determined in accordance with DIN 22022-2:2001–02.

#### 2.2.4 pH and energy content measurement

The pH of PM and CPH was measured in accordance with the TMECC 04–11-A standard, following the procedure described by Saba et al. (2023). The higher heating value (HHV) and lower heating value (LHV) were determined using an adiabatic bomb calorimeter in compliance with the DIN EN ISO 21654 (2021–12) standard.

### 2.3 Biogas potential assessment

#### 2.3.1 Experimental design

Batch AD tests were conducted in accordance with the VDI 4630 standard (VDI 4630, 2016) to quantify the biogas potential of PM and CPH and to assess the effects of substrates’ carbon-to-nitrogen (C/N) ratio and temperature regime on anaerobic co-digestion performance. The batch assays were performed using the ANKOM gas production system. Digestion was carried out in 500 mL glass reactors with a working volume of 400 mL, loaded at a substrate concentration of 10 g VS/L and a substrate-to-inoculum (S/I) ratio of 0.5 on a VS basis. Reactors were hermetically sealed and incubated for 30 days in thermostatically controlled water baths at 37 °C (mesophilic) or 55 °C (thermophilic). To prevent sedimentation and scum formation, reactors were stirred daily for 1 min using a magnetic stirrer.

**FIGURE 1:**
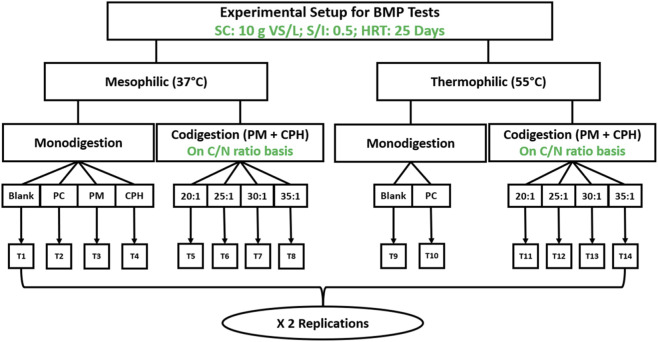
Experimental design of batch AD assays under mesophilic and thermophilic conditions for mono- and co-digestion of PM and CPH at varying C/N ratios.

Mono-digestion tests of PM and CPH were conducted under mesophilic conditions to establish baseline biogas potentials and ensure comparability with widely reported batch AD literature. Co-digestion experiments were designed using a two-factorial approach combining four targeted C/N ratios (20, 25, 30, and 35) and two temperature regimes (37 °C and 55 °C). Substrate mixing ratios were calculated based on volatile solids content and elemental carbon and nitrogen concentrations, using Equation 4, adapted from Haider et al. (2015), to achieve the desired C/N ratio while maintaining a constant organic loading. Each treatment was conducted in duplicate. Control assays included blank tests containing inoculum only, used to correct for background biogas production, and positive control tests using microcrystalline cellulose to verify inoculum activity and system integrity, as recommended by VDI 4630. In total, 28 batch AD assays were performed, comprising mono-digestion, co-digestion, blank, and positive control tests, enabling robust evaluation of main and interaction effects of substrate composition and temperature. The overall experimental design is illustrated in Figure 1.

$$C/N=\frac{\left(Q_{{\text{VS}}_{\text{PM}}}\times C_{\text{PM}}\right)+\left(Q_{{\text{VS}}_{\text{CPH}}}\times C_{\text{CPH}}\right)}{\left(Q_{{\text{VS}}_{\text{PM}}}\times N_{\text{PM}}\right)+\left(Q_{{\text{VS}}_{\text{CPH}}}\times N_{\text{CPH}}\right)}$$ (4)

where: $Q_{{\text{VS}}_{\text{PM}}}$ and $Q_{{\text{VS}}_{\text{CPH}}}$ , the amounts of volatile solids from PM and CPH, respectively (g), $C_{\text{PM}}$ and $C_{\text{CPH}}$ , the carbon content of PM and CPH, respectively (%), $N_{\text{PM}}$ and $N_{\text{CPH}}$ , the nitrogen content of PM and CPH, respectively (%).

### 2.4 Statistical analysis methods

All biogas potential data were analyzed using descriptive statistics, including mean and standard deviation (SD), and analysis of variance (ANOVA) to assess the effects of C/N ratio, temperature regime, and their interaction on cumulative biogas yield. Assumptions of normality and homogeneity of variances were evaluated using Shapiro-Wilk and Levene’s tests, respectively. In cases of heteroscedasticity, Welch-type ANOVA and heteroscedasticity-consistent (HC3) robust general linear models were applied to ensure reliable inference. Significant effects were further explored using Tukey’s HSD *post hoc* test at a 95% confidence level.

## 3 Results and discussion

### 3.1 Results

#### 3.1.1 Substrate characterization

PM and CPH exhibited complementary physicochemical properties, as presented in Table 1. PM was alkaline (pH 8.70 ± 0.02), whereas CPH was near neutral (pH 6.89 ± 0.04). Total solids exceeded 94% (FM basis) for both substrates, with PM showing higher ash content (26.82% ± 0.26% TS) than CPH (11.35% ± 0.97% TS). Volatile solids content was substantially higher in CPH (88.65% ± 0.97% TS) compared with PM (73.18% ± 0.26% TS).

**TABLE 1:** Physicochemical properties of PM and CPH.

Parameter	Unit	CPH	PM
pH	-	6.89 ± 0.04	8.70 ± 0.02
TS	%FM	94.82 ± 0.93	96.34 ± 0.48
MC	%FM	5.18 ± 0.93	3.66 ± 0.48
VS	%TS	88.65 ± 0.97	73.17 ± 0.26
AC	%TS	11.35 ± 0.97	26.82 ± 0.26
Lignin	%TS	23.71 ± 0.48	9.90 ± 0.53
Cellulose	%TS	26.59 ± 0.70	25.02 ± 0.65
Hemicellulose	%TS	8.32 ± 0.61	12.15 ± 0.79
Carbon (C)	%TS	48.60 ± 0.10	35.15 ± 0.27
Hydrogen (H)	%TS	5.34 ± 0.08	4.41 ± 0.07
Oxygen (O)	%TS	38.25 ± 0.05	30.05 ± 0.53
Nitrogen (N)	%TS	0.95 ± 0.14	2.19 ± 0.06
Sulfur (S)	%TS	0.10 ± 0.01	0.35 ± 0.03
C/N	-	51.16	16.07
Iron (Fe)	%TS	0.017 ± 0.003	0.26 ± 0.09
Calcium (Ca)	%TS	0.84 ± 0.01	3.76 ± 0.19
Potassium (K)	%TS	4.15 ± 0.02	2.53 ± 0.15
Magnesium (Mg)	%TS	0.41 ± 0.01	0.48 ± 0.09
Sodium (Na)	%TS	0.003 ± 0.001	0.46 ± 0.06
Phosphorus (P)	%TS	0.14 ± 0.01	1.49 ± 0.07
HHV	kJ/kg	16,987.00 ± 6.56	13,187 ± 50.51
LHV	kJ/kg	15,824.00 ± 22.00	12,227 ± 65.51

Elemental analysis indicated that CPH was carbon-rich (48.60% ± 0.10% TS) with a high C/N ratio (51.16), while PM contained higher nitrogen (2.19% ± 0.06% TS) and a lower C/N ratio (16.07). Fiber fractionation showed a higher lignin content in CPH (23.71% ± 0.48% TS) than in PM (9.90% ± 0.53% TS). PM contained higher concentrations of inorganic nutrients (Ca, P, Na, Fe, S). Higher and lower heating values were greater for CPH (16.99 ± 0.01 and 15.82 ± 0.02 MJ/kg, respectively) than for PM (13.19 ± 0.05 and 12.23 ± 0.07 MJ/kg).

#### 3.1.2 Mono-digestion performance

**FIGURE 2:**
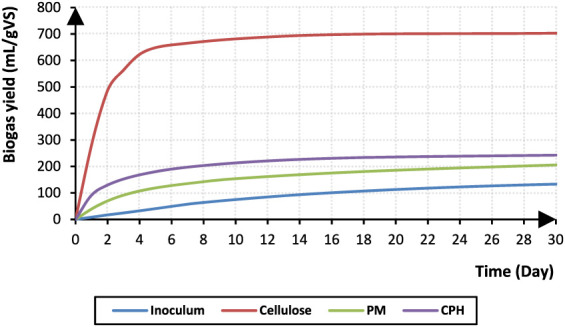
Cumulative biogas production profile.

**FIGURE 3:**
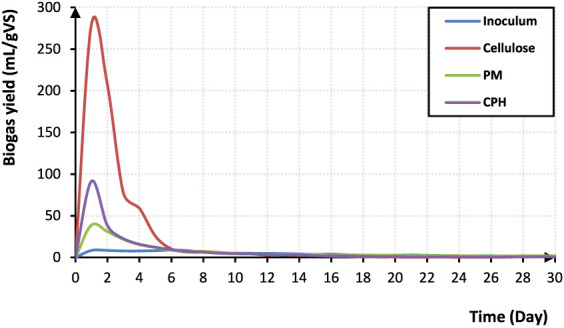
Daily biogas production yield.

**FIGURE 4:**
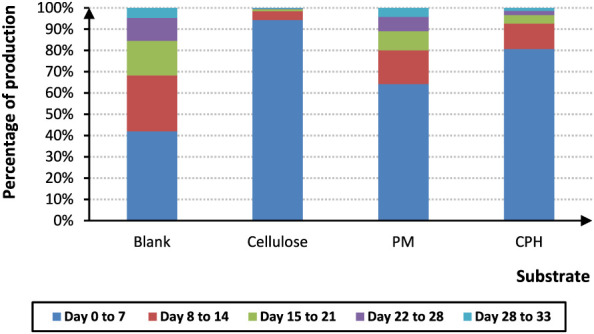
Weekly biogas production percentage per substrate.

As presented in Figure 2, under mesophilic conditions (37 °C), mono-digestion of CPH yielded a cumulative biogas production of 244.65 ± 8.48 mL/g VS, exceeding that of PM (210.73 ± 27.12 mL/gVS). The positive control (cellulose) reached 705.46 ± 13.98 mL/g VS, while the inoculum blank produced 136.69 ± 6.90 mL/g VS. Daily (Figure 3) and weekly (Figure 4) production profiles showed that CPH generated approximately 80.6% of its cumulative biogas within the first 7 days, followed by a rapid decline and stabilization after day 21. In contrast, PM exhibited slower initial production, with 64.2% of total biogas generated in the first week, and a more evenly distributed pattern over the digestion period. Daily production profiles reflected an early sharp peak for CPH and a more sustained, lower-amplitude pattern for PM.

#### 3.1.3 Co-digestion performance

**FIGURE 5:**
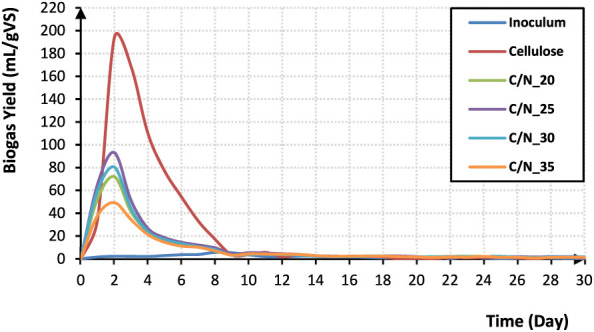
Co-digestion mesophilic daily biogas yield (mL/g VS).

**FIGURE 6:**
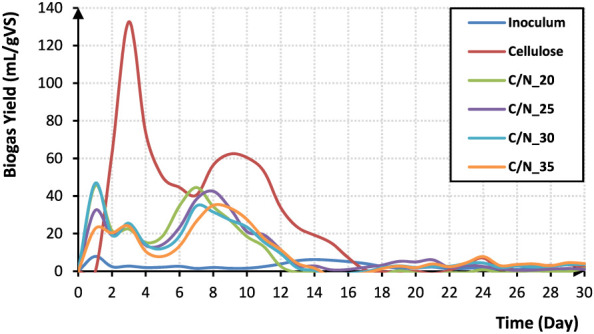
Co-digestion thermophilic daily biogas yield (mL/g VS).

As displayed in Figures 5, 6, daily biogas production during co-digestion varied substantially with both C/N ratio and temperature. Under mesophilic conditions (Figure 6), gas production peaked rapidly within the first 3 days, particularly at C/N 25 and C/N 30, reaching maximum daily yields of 93.16 and 80.46 mL/g VS, respectively, on day 2. Daily yields then declined progressively across all treatments, with C/N 35 consistently showing the lowest production. Under thermophilic conditions (Figure 6), daily net biogas production profiles were less stable and exhibited pronounced fluctuations, including mid-phase depressions and occasional negative blank-corrected values, particularly at C/N 20 and C/N 35. These negative values occurred when daily gas production from the inoculum control exceeded that of the corresponding substrate reactors, resulting in temporary net subtraction after background correction. In contrast, treatments at C/N 25 and C/N 30 maintained comparatively sustained and more regular production throughout the digestion period.

**FIGURE 7:**
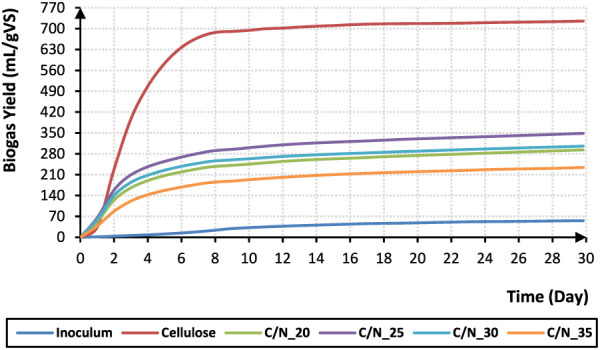
Co-digestion mesophilic cumulative biogas yield (mL/g VS).

**FIGURE 8:**
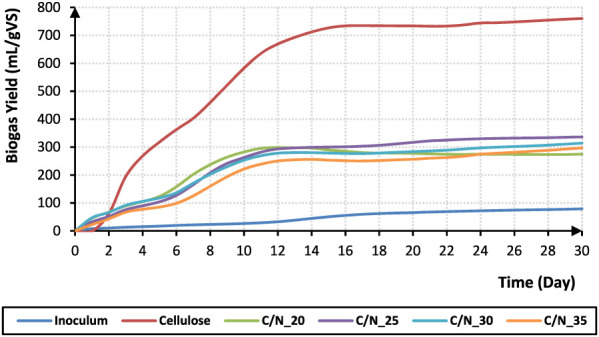
Co-digestion thermophilic cumulative biogas yield (mL/g VS).

Cumulative biogas yields under mesophilic conditions (Figure 7) followed the order: C/N 25 (348.65 ± 10.44 mL/g VS) > C/N 30 (306.02 ± 18.62 mL/g VS) > C/N 20 (293.21 ± 35.43 mL/g VS) > C/N 35 (234.15 ± 13.05 mL/g VS). Under thermophilic conditions (Figure 8), the highest yield was again observed at C/N 25 (336.21 ± 15.92 mL/g VS), followed by C/N 30 (314.15 ± 17.89 mL/g VS), C/N 35 (296.98 ± 10.86 mL/g VS), and C/N 20 (275.02 ± 13.71 mL/g VS).

#### 3.1.4 Statistical analysis results

A two-way analysis of variance (ANOVA) was performed to assess the effects of C/N ratio, temperature regime, and their interaction on cumulative biogas yield. Prior to model fitting, ANOVA assumptions were evaluated. As summarized in Table 2, the Shapiro-Wilk test indicated that the model residuals were normally distributed (W = 0.972, p = 0.865). In contrast, Levene’s test revealed a pronounced violation of the homogeneity of variance assumption (F = 1.11 ✕ 10^29^, p < 0.001), indicating substantial heteroscedasticity among treatment groups. The observed heteroscedasticity likely reflects genuine process-dependent variability arising from unstable thermophilic responses at the nitrogen-rich and carbon-rich extremes, differential ammonia inhibition in PM-dominant mixtures, and non-uniform hydrolysis of lignocellulosic CPH fractions.

**TABLE 2:** Normality and Homogeneity Assumptions Test results.

Test	Df	W Statistic	p-value	Interpretation
Shapiro-wilk test (Normality)	8	0.972	0.8652	Residuals are normally distributed
Levene’s test (homogeneity of variance)	8	1,11 × 10^29^	<0.001	Homogeneity of variances is violated

Results of the classical two-way ANOVA are presented in Table 3. The C/N ratio exerted a highly significant effect on cumulative biogas yield (F = 16.17, p = 0.001). The main effect of temperature was not statistically significant (F = 1.48, p = 0.259). However, a significant interaction between C/N ratio and temperature was detected (F = 4.96, p = 0.031), demonstrating that the influence of substrate composition varied across thermal regimes.

**TABLE 3:** Analyses of variance results.

Effect	ANOVA	Welch-type ANOVA	HC3-robust GLM
C/N Ratio	F = 16.17	F = 68.75	F = 37.58
	p = 0.001	p < 0.001	p < 0.001
Temperature	F = 1.48	F = 38.51	F = 0.26
	p = 0.259	p = 0.0003	p = 0.624
C/N × temperature	F = 4.96	F = 12.37	F = 6.18
	p = 0.031	p = 0.002	p = 0.018

To account for the detected heteroscedasticity, the analysis was further validated using two variance-robust statistical approaches (Table 3). First, a Welch-type ANOVA based on weighted least squares revealed significant effects for all factors, including C/N ratio (F = 68.75, p < 0.001), temperature (F = 38.51, p = 0.0003), and their interaction (F = 12.37, p = 0.002). Second, a heteroscedasticity-consistent general linear model (HC3-robust GLM) confirmed the significance of the C/N ratio (F = 37.58, p < 0.001) and the C/N × temperature interaction (F = 6.18, p = 0.018), while the temperature main effect remained non-significant (F = 0.26, p = 0.624). This consistency between the classical ANOVA and the HC3-robust model indicates that the effect of temperature is primarily expressed through its interaction with nutrient balance rather than as an independent driver.

Post-hoc comparisons using Tukey’s Honest Significant Difference (HSD) test identified five statistically significant pairwise differences (p < 0.05) (Table 4). In particular, the C/N 25 treatment under mesophilic conditions produced significantly higher cumulative biogas yields than C/N 20 under thermophilic conditions and C/N 35 under mesophilic operation. In addition, both C/N 25 and C/N 30 treatments, irrespective of temperature regime, significantly outperformed C/N 35 under mesophilic conditions, which consistently yielded the lowest biogas production.

**TABLE 4:** Tukey HSD Post-hoc Test results.

Group 1	Group 2	Mean difference (mL/g VS)	Adjusted p-value	Significant p < 0.05
C/N 25 (mesophilic)	C/N 35 (mesophilic)	114.506	0.002	Yes
C/N 25 (mesophilic)	C/N 20 (thermophilic)	73.633	0.027	Yes
C/N 25 (thermophilic)	C/N 35 (mesophilic)	102.062	0.004	Yes
C/N 30 (thermophilic)	C/N 35 (mesophilic)	80.006	0.017	Yes
C/N 30 (mesophilic)	C/N 35 (mesophilic)	71.873	0.031	Yes

Across all statistical approaches, the C/N ratio emerged as the most robust determinant of cumulative biogas yield, with a statistically significant interaction with temperature regime. While the Welch-type ANOVA suggested a potential independent contribution of temperature, both the classical ANOVA and the conservative HC3-robust model indicate that temperature predominantly modulates digestion performance through its interaction with substrate composition. Notably, C/N 25 under mesophilic conditions was consistently identified as the highest-performing configuration across all models and *post hoc* comparisons.

### 3.2 Discussion

#### 3.2.1 Assessment of substrate suitability for biogas production

The physicochemical characterization confirmed that both CPH and PM possess complementary properties, making them suitable for co-digestion. The pH values (Table 1) revealed a potential buffering synergy. CPH was slightly acidic (pH 6.89) due to its lignocellulosic and phenolic nature (Meza-Sepulveda et al., 2024), while PM was alkaline (pH 8.70) owing to its high ammoniacal nitrogen and bicarbonate content (Kacprzak et al., 2023; Swelum et al., 2021). This contrast suggests that their combination could stabilize pH within the optimal methanogenic range (6.8–7.4).

In terms of biodegradability, CPH exhibited higher volatile solids (88.65% TS) compared to PM (73.18% TS), indicating greater organic potential, although PM’s higher ash content (26.82% TS) reflects more inert mineral matter that can dilute the effective organic fraction. This aligns with literature values where lignocellulosic by-products, such as cocoa husk, typically contain around 80%–90% VS/TS (Antwi et al., 2019; Nakhate et al., 2022), compared to 65%–75% for poultry or cattle manure (Jurgutis et al., 2020; Wang, 2014). Fiber composition analysis revealed that CPH contained more lignin (23.71% TS), which could hinder hydrolysis, whereas PM had a greater hemicellulose content (12.15% TS), promoting rapid initial degradation. The grinding and drying pretreatment applied in this study likely improved substrate digestibility by reducing particle size and weakening lignin bonds, as supported by the literature, which indicates a 15%–25% improvement in methane yield, after similar pretreatments, under mesophilic digestion (Díaz-González et al., 2022; Ouattara et al., 2021).

Elemental analysis also revealed contrasting levels of carbon and nitrogen (Table 1). CPH was carbon-rich (C/N = 51.16), and PM was nitrogen-rich (C/N = 16.07), both of which fell outside the optimal 20–30 range (Shahbaz et al., 2020; Wang et al., 2014). This explains their limited mono-digestion performance and highlights the importance of blending to achieve a nutrient balance. The ANOVA confirmed that the C/N ratio is a highly significant determinant of yield (p < 0.001), demonstrating that co-digestion effectively mitigates nitrogen deficiency and ammonia inhibition. Moreover, PM provided essential micronutrients such as phosphorus, calcium, magnesium, and sodium, which support enzymatic activity and methanogenesis when maintained within tolerable levels (Czatzkowska et al., 2020; Liebetrau and Pfeiffer, 2020; Somak and Sagar, 2023). Finally, energy characterization showed that CPH possessed higher heating values (HHV = 16,987 kJ/kg; LHV = 15,824 kJ/kg) than PM (HHV = 13,187 kJ/kg; LHV = 12,227 kJ/kg), confirming its role as the primary energy source. Altogether, these findings establish that co-digestion of CPH and PM creates physicochemical and nutritional complementarity, balancing acidity, nutrient supply, and energy density, thereby enhancing biodegradability, microbial stability, and overall biogas productivity compared to mono-digestion.

#### 3.2.2 Mono-digestion performance

##### 3.2.2.1 Experimental system validation

The positive control provided a strong validation of the experimental system. Cellulose yielded 705.46 ± 13.98 mL/g VS. This value closely aligns with the German VDI 4630 (2016) guideline, which reports a standard biogas potential for cellulose of 671–820 mL/g VS (745% ± 10%) under mesophilic conditions. The results confirm the inoculum’s enzymatic activity and microbiological efficiency, ruling out inhibitory conditions or operational faults. Thus, the observed differences in yields between PM and CPH can be confidently attributed to substrate-specific properties rather than methodological artifacts. It should nevertheless be acknowledged that the inoculum originated from a stable mesophilic agricultural digester in Germany rather than from a locally adapted West African anaerobic system. Anaerobic microbial consortium obtained in Germany may operationally differ from an indigenous microbial consortium in West African context. Studies show that inoculum origin primarily affects substrate degradation rates and lag phases rather than the ultimate specific biogas yield for many substrates, although substrate-adapted inocula can accelerate conversion of specific materials (e.g., cellulose, sewage sludge, etc.) (Koch et al., 2017). Geographic differences in feedstocks, ambient climatic conditions, and biogas plant operation practices can lead to differences in digestate microbial community composition between German and West African biogas plant digesters (Dai et al., 2023; Favale et al., 2025). This may influence initial hydrolysis and acidogenesis rates and cause longer adaptation when inoculum and substrate are mismatched. Therefore, the biogas yield results here report the potential of the substrates under a standard mesophilic inoculum, and it should be noted that in-field performance in West African digesters could differ in biogas production rate and start-up behaviour (Chen et al., 2024; Niya et al., 2024; Pinto et al., 2025).

##### 3.2.2.2 Biogas potential of CPH and PM

The mono-digestion performance of CPH and PM revealed important insights into their biodegradability and suitability for AD under mesophilic conditions. As shown in Figure 2, CPH achieved a higher cumulative yield (244.65 ± 8.48 mL/g VS) than PM (210.73 ± 27.12 mL/g VS), reflecting its higher volatile solids content (84.06% FM, VS/TS = 0.89) and energy density (HHV = 16,987 kJ/kg). However, despite its higher organic fraction, CPH exhibited an early production peak followed by a rapid plateau (Figure 3). This profile reflects its high lignin content (23.71% TS), which limits microbial hydrolysis and prevents complete conversion of structural carbohydrates. Lignin recalcitrance has been identified as a significant constraint in lignocellulosic digestion (Liebetrau and Pfeiffer, 2020; Nakhate et al., 2022). The initial burst of gas production likely arose from easily hydrolyzable fractions (soluble sugars and hemicellulose), partly enhanced by the applied physical pretreatment (drying and fine grinding). However, without chemical, biological, or other advanced pretreatments, the lignin-rich fraction remained resistant, resulting in incomplete utilization of CPH’s theoretical potential.

The CPH’s biogas yield obtained in this study (244.65 ± 8.48 mL/g VS) was lower than values reported in previous studies. For example, Hennessey-Ramos et al. (2024) recorded a yield of 314.86 ± 4.45 mL/g VS under a 35-day mesophilic (35 °C) batch digestion, using inoculum from an active anaerobic digester with an S/I ratio of 1/3. While, Antwi et al. (2019) reported an even higher value of 357 mL/g VS, recorded under similar conditions (mesophilic at 38 °C, inoculum from an active biodigester, and a S/I ratio of 1/3). These discrepancies likely arise from geographic variation in CPH composition, the absence of advanced pretreatment in the present study, and differences in inoculum type, particularly the feeding material of the active anaerobic digester from which the inoculum was sourced. Notably, the absence of chemical or thermal pretreatment in this study likely limited biodegradation of lignin-rich sections, which are known to hinder complete hydrolysis of lignocellulosic materials such as CPH.

By contrast, PM demonstrated a more sustained gas production profile, consistent with its moderate volatile solids content (VS of 70.50% and VS/TS ratio of 0.73) and high inorganic fraction. As shown in Figure 3, PM’s lower cumulative yield (210.73 ± 27.12 mL/g VS) compared to CPH is likely due to dilution from its high ash content (25.84%), which reduces the proportion of organic matter available for conversion. Moreover, PM’s relatively low C/N ratio (16.07) may have contributed to increased ammonia levels during digestion. A low C/N ratio may lead to excessive nitrogen loading, which is known to inhibit methanogenesis through free ammonia toxicity, especially under conditions of poor buffering (Duan et al., 2018; Haider et al., 2015; Wang et al., 2014). Despite these challenges, PM maintained a stable biogas production rate over time, likely due to its rich supply of essential nutrients, including phosphorus, magnesium, and calcium. These micronutrients are important for microbial growth and enzyme function, as noted by Zhu et al. (2023).

The 210.73 ± 27.12 mL/g VS yield for PM was also lower than that reported for many other values. For instance, Al-Zoubi et al. (2024) reported 285 mL/g VS under a 30-day mesophilic digestion (37 °C), considering a S/I ratio of 1/2. Michailidou et al. (2024) observed yields ranging from 363 to 400 mL/g VS under mesophilic conditions, using cattle manure as the inoculum and the AMPTS III system. Even higher yields were recorded by Jurgutis et al. (2020) (508 mL/g VS), using the AMPTS II system with an active digestate as inoculum at an S/I ratio of 1/2, and Li et al. (2013) (617 mL/g VS), considering a substrate concentration of 3 g VS/L and an S/I ratio of 1/2, both under mesophilic conditions. It is important to note, however, that differences in inoculum loading may partly explain these variations. In the present work, a relatively high inoculum-to-substrate ratio was deliberately used to maintain inoculum dominance, ensure adequate microbial biomass, and provide sufficient buffering capacity throughout batch AD assays. By contrast, lower inoculum proportions in other studies may alter the hydrolysis–methanogenesis balance, leading to different biogas conversion dynamics. Therefore, direct comparison of absolute yields should be interpreted with caution, as biogas productivity is influenced not only by substrate characteristics but also by reactor configuration and inoculum loading strategy. Despite the relatively low C/N ratio of PM (16.07), no abrupt production collapse or severe lag phase was observed, suggesting that the selected inoculum loading was sufficient to maintain stable digestion conditions. Consequently, the comparatively modest PM yield observed in this study is more plausibly linked to substrate heterogeneity, elevated inorganic matter, and the recalcitrant lignocellulosic contribution of bedding residues than to major process inhibition. The higher standard deviation recorded for PM (±27.12 mL/g VS), compared with CPH (±8.48 mL/g VS), further reflects the intrinsic compositional variability of poultry manure. Nevertheless, the sustained gas production beyond day 14 confirms its microbial compatibility and supports its role as a stabilizing co-substrate in anaerobic co-digestion.

#### 3.2.3 Co-digestion performance assessment

##### 3.2.3.1 Biogas yield improvement

The results clearly demonstrate that anaerobic co-digestion of PM and CPH substantially enhances biogas production compared with mono-digestion of either substrate, confirming strong synergistic effects. Across all tested mixtures, co-digestion consistently outperformed mono-digestion, indicating that combining nitrogen-rich and carbon-dense substrates creates more favorable biochemical conditions for anaerobic microbial consortia. This synergy primarily arises from nutrient complementarity: PM, characterized by a low C/N ratio, high nitrogen content, and significant buffering capacity, effectively compensates for CPH’s carbon-rich, lignocellulosic, and slightly acidic nature. As a result, co-digestion promotes pH stability, balanced nutrient availability, and improved enzymatic activity, thereby facilitating more efficient hydrolysis, acidogenesis, and methanogenesis.

Beyond nutrient balancing, co-digestion may improve process stability by reducing the likelihood of inhibitory conditions commonly associated with mono-digestion of individual substrates. Poultry manure, characterized by a relatively low C/N ratio, is generally associated with higher nitrogen availability, which under anaerobic conditions may lead to increased ammonia formation and potential inhibition of methanogenic activity. In contrast, cocoa pod husks, due to their lignocellulosic nature, exhibit slower hydrolysis kinetics, leading to transient accumulation of intermediate metabolites, such as volatile fatty acids (VFAs), when degradation rates exceed methanogenic conversion capacity. In this context, co-digestion of both substrates promotes a more balanced biochemical environment by combining complementary degradation characteristics: the readily available nutrients from PM support microbial growth, while the more recalcitrant fraction of CPH moderates rapid substrate conversion, thereby contributing to process stability. This synergy reduces the likelihood of severe imbalance between acidogenic and methanogenic phases. Statistical analysis supports the importance of substrate composition, as the two-way ANOVA identified the C/N ratio as a significant factor influencing cumulative biogas yield (p < 0.001), whereas temperature alone had no significant independent effect. However, the significant interaction between C/N ratio and temperature (p = 0.031) suggests that thermal effects are substrate-dependent and secondary to overall substrate stoichiometry, reinforcing the importance of feedstock composition in determining process performance.

Quantitatively, co-digestion yielded substantial improvements in yield relative to mono-digestion benchmarks. While PM and CPH mono-digestion yielded 210.73 ± 27.12 mL/g VS and 244.65 ± 8.48 mL/g VS, respectively (Figure 2), all co-digestion scenarios (Figure 7) achieved higher or comparable performance, except for the most carbon-rich mixture. The highest biogas yield was obtained at a C/N ratio of 25 (348.65 ± 10.44 mL/g VS), corresponding to a 65.45% increase over PM and a 42.51% increase over CPH, as presented in Table 5. The C/N 30 mixture also showed marked improvements (45.22% and 25.08% relative to PM and CPH, respectively), confirming that the optimal nutrient window lies between C/N 25 and 30. In contrast, the C/N 35 condition yielded only a marginal improvement over PM (11.11%) and underperformed CPH (−4.29%), reflecting nitrogen limitation and the inhibitory effect of lignin-rich fractions in CPH. These results illustrate that co-digestion alone is insufficient to guarantee performance gains unless substrate ratios are carefully optimized. These observed improvement rates are consistent with previous studies reporting enhanced methane or biogas yields from PM-based co-digestion systems. Awais et al. (2016) demonstrated significant yield enhancement when PM was co-digested with crop residues under mesophilic conditions, attributing the improvement to a more balanced nutrient profile and greater microbial stability. Similarly, Dahunsi et al. (2019) reported yield increases of up to 68% when PM was co-digested with alkaline-pretreated CPH, highlighting the combined benefits of nutrient complementarity and improved lignocellulosic accessibility. Similarly, Rahman et al. (2023) observed a 16% increase in yield when PM was co-digested with kitchen waste under mesophilic conditions, confirming that nutrient balancing through co-digestion consistently improves yields across diverse organic substrates. Although no advance pretreatment methods were used in the present study, the physical pretreatment steps (oven-drying and fine grinding) likely improved hydrolysis by increasing the substrate surface area and partially disrupting the lignin-cellulose matrix, thereby reinforcing the observed synergistic effects.

**TABLE 5:** Percentage improvement in biogas yield compared to mono-digestion.

Condition	Cumulative yield (mL/g VS)	% improvement vs. PM	% improvement vs. CPH
Mono-digestion: PM	210.73 ± 27.12	-	-
Mono-digestion: CPH	244.65 ± 8.48	16.10%	-
Co-digestion C/N 20	293.21 ± 35.43	39.14%	19.85%
Co-digestion C/N 25	348.65 ± 10.44	65.45%	42.51%
Co-digestion C/N 30	306.02 ± 18.62	45.22%	25.08%
Co-digestion C/N 35	234.15 ± 13.05	11.11%	−4.29%

Overall, these findings demonstrate that co-digestion of PM and CPH significantly improves biogas yield when appropriate substrate ratios are applied, with C/N ratios of 25–30 providing the most favorable balance for microbial activity and process stability. The results underscore that nutrient stoichiometry, rather than temperature alone, is the primary driver of enhanced performance. From a practical perspective, this highlights co-digestion as a robust, low-cost strategy for maximizing energy recovery from heterogeneous agricultural residues, particularly in decentralized biogas systems where feedstock quality and availability vary.

##### 3.2.3.2 Combined effects of C/N ratio and temperature on co-digestion performance

**FIGURE 9:**
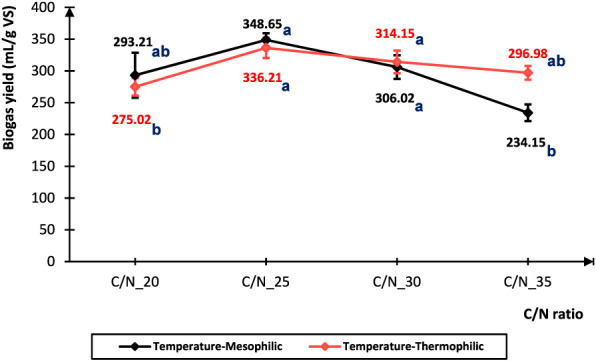
Comparative cumulative biogas yields obtained from PM–CPH co-digestion under mesophilic (37 °C) and thermophilic (55 °C) conditions at varying C/N ratios (20, 25, 30, and 35). *Bars represent mean cumulative biogas production, while error bars indicate ± standard deviation of duplicate assays (n = 2). Different superscript letters denote statistically significant differences among treatments, as determined by Tukey’s HSD test (p < 0.05)*.

The carbon-to-nitrogen (C/N) ratio and operating temperature are among the most influential parameters governing anaerobic co-digestion performance, as they jointly regulate microbial metabolism, enzymatic activity, and tolerance to inhibitory compounds (Ibro et al., 2022; Shahbaz et al., 2020). In this study, systematic variation of the C/N ratio (20, 25, 30, and 35) under mesophilic (37 °C) and thermophilic (55 °C) conditions revealed that nutrient balance was the primary determinant of biogas yield, while temperature played a secondary, modulatory role. This conclusion is strongly supported by the statistical analysis, which found the C/N ratio to be highly significant (p < 0.001), whereas temperature alone was not (p = 0.259). However, the significant interaction between C/N ratio and temperature (p = 0.031) demonstrates that thermal effects are substrate-dependent and cannot be optimized independently of nutrient stoichiometry. This combined effect of C/N ratio and temperature is illustrated in Figure 9.

**FIGURE 10:**
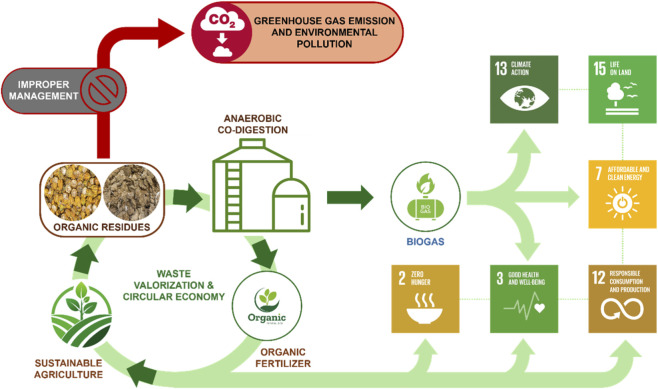
Conceptual framework illustrating the synergistic benefits of PM and CPH co-digestion and its linkages to relevant Sustainable Development Goals (SDGs).

Across both temperature regimes, the C/N ratio of 25 consistently yielded the highest cumulative biogas (Figure 10), indicating this ratio as the optimal balance for PM-CPH co-digestion. At this ratio, yields reached 348.65 ± 10.44 mL/g VS under mesophilic conditions and 336.21 ± 15.92 mL/g VS under thermophilic conditions. This performance reflects an optimal equilibrium between carbon availability for energy generation and nitrogen supply for microbial growth and enzyme synthesis, enabling efficient progression through hydrolysis, acidogenesis, and methanogenesis. These results align well with the broadly reported optimal C/N window of 20–30 for mixed organic substrates (Choi et al., 2020; Shahbaz et al., 2020; Wang et al., 2014). The stability and high productivity observed at C/N 25, particularly under mesophilic conditions (Figures 6, 8), further indicate that the microbial community operated without carbon limitation, nitrogen overload, or significant accumulation of inhibitory intermediates.

At lower C/N ratios, particularly C/N 20, biogas yields declined under both temperature regimes, with the effect being more pronounced under thermophilic conditions. The nitrogen-rich nature of this mixture likely increased ammoniacal nitrogen concentrations, shifting the NH_4_^+^/NH_3_ equilibrium toward free ammonia (NH_3_), especially at elevated temperatures. Free ammonia readily diffuses across microbial cell membranes and disrupts methanogenic pathways, leading to reduced yields and unstable kinetics (Chen et al., 2008; Yang et al., 2025; Yenigün and Demirel, 2013). This mechanism explains the lower yields at C/N 20 (293.21 ± 35.43 mL/g VS mesophilic; 275.02 ± 13.71 mL/g VS thermophilic) and the irregular daily production patterns observed under thermophilic operation, as displayed in Figures 6, 8. Similar ammonia-related inhibition under thermophilic conditions has been widely reported in manure-rich digestion systems (Duan et al., 2018; Haider et al., 2015), reinforcing the sensitivity of nitrogen-rich substrates to high-temperature operation.

Conversely, at higher C/N ratios, particularly C/N 35, nitrogen limitation and increased lignocellulosic content constrained biogas production under mesophilic conditions, resulting in the lowest yield of the study (234.15 ± 13.05 mL/g VS). The high CPH proportion increased the lignin load, thereby limiting hydrolysis and slowing overall digestion kinetics. Under thermophilic conditions, however, this limitation was partially alleviated, with yields increasing to 296.98 ± 10.86 mL/g VS. Elevated temperatures enhance hydrolytic enzyme activity and accelerate the breakdown of structurally complex, carbon-rich substrates, thereby improving accessibility of fermentable compounds. This behavior is consistent with previous studies reporting superior degradation of lignocellulosic feedstocks such as straw and crop residues at 55 °C compared to 37 °C (David et al., 2018; Singh et al., 2023). These findings indicate that thermophilic digestion can be advantageous for carbon-rich, recalcitrant mixtures, provided that nitrogen availability and buffering capacity are sufficient.

The intermediate C/N 30 configuration exhibited robust, relatively stable performance across both temperature regimes, with yields of 306.02 ± 18.62 mL/g VS (mesophilic) and 314.15 ± 17.89 mL/g VS (thermophilic). This suggests that the PM-CPH system exhibits some resilience within a broader optimal C/N window (25–30), which is particularly relevant for practical applications where feedstock composition may fluctuate. Kinetic profiles further support this interpretation: mesophilic digestion at C/N 25 and 30 was characterized by rapid early gas production followed by a smooth decline, indicative of well-buffered and stable microbial activity (Figure 5). In contrast, thermophilic digestion exhibited greater variability, including mid-phase drops and fluctuations at suboptimal C/N ratios, reflecting a narrower stability margin under high-temperature operation (Figure 6).

#### 3.2.4 Practical implications for sustainable waste management and bioenergy deployment

The results of this study demonstrate that anaerobic co-digestion of PM and CPH represents a technically robust and operationally realistic waste-to-energy strategy for agriculture-based economies. By prioritizing substrate balancing rather than energy-intensive process intensification, the optimized mesophilic configuration (C/N 25–30) achieved high and stable biogas production while maintaining relatively low operational complexity. Such characteristics are particularly advantageous for regions where agricultural residues are abundant but access to advanced process monitoring, pretreatment technologies, and continuous energy inputs remains limited. The ability to enhance biogas productivity without chemical buffering, sophisticated pretreatment, or thermophilic heating requirements suggests that performance optimization can be achieved primarily through feedstock management and substrate complementarity.

From a technology-readiness perspective, the investigated system corresponds approximately to TRL 4–5 (Salvador-Carulla et al., 2024), since the process was evaluated under controlled laboratory conditions using standardized BMP methodologies (VDI 4630, 2016) and commercially available analytical equipment. Nevertheless, the observed process stability under mesophilic conditions provides a realistic pathway toward pilot-scale continuous operation (TRL 6–7), particularly for decentralized agricultural applications. Compared with thermophilic digestion, mesophilic operation generally provides greater process stability, lower parasitic heating demand, and higher tolerance to operational disturbances, characteristics that are particularly advantageous for decentralized and small-scale biogas systems operating under resource-constrained conditions (Steiniger et al., 2023). Beyond renewable energy generation, PM-CPH co-digestion presents important environmental and agronomic co-benefits. Controlled anaerobic stabilization of poultry manure reduces uncontrolled methane emissions, ammonia volatilization, odor generation, and pathogen dissemination associated with conventional manure disposal practices (Swelum et al., 2021). Simultaneously, valorization of cocoa pod husks diverts lignocellulosic residues from open burning and uncontrolled decomposition, thereby reducing particulate emissions and environmental contamination in cocoa-producing areas (Ouattara et al., 2021). In addition, digestate reuse provides a nutrient-rich organic amendment capable of supporting soil fertility restoration and reducing dependence on synthetic fertilizers. Such integrated resource recovery pathways are increasingly recognized as central components of climate-smart agriculture and circular bioeconomy systems (FAO, 2022).

The optimized PM-CPH configuration also demonstrates strong relevance for decentralized rural energy systems. A practical feedstock-to-energy extrapolation indicates that approximately 100t VS/year of recoverable PM could be co-digested with nearly 118t VS/year of recoverable CPH under the optimum mesophilic C/N ratio of 25, corresponding to a total annual substrate throughput of approximately 218t VS. Based on the maximum biogas yield obtained in this study (348.65m3 Biogas/t VS), such a substrate stream could theoretically generate nearly 7.6 ✕ 104 m^3^ of biogas annually. Using a conservative electricity conversion factor of 1.8 kWh/m^3^ biogas, consistent with values commonly reported for decentralized biogas-based power generation systems (Jaman et al., 2022; Scarlat et al., 2018), the optimized substrate stream could theoretically generate approximately 136.8 MWh of electrical energy annually. This level of energy production highlights the potential of relatively modest biomass aggregation to support decentralized energy services for rural households, small agro-processing enterprises, and community-scale productive activities. Such decentralized biogas systems are increasingly recognized as viable solutions for improving rural energy access, strengthening agricultural residue valorization, and promoting sustainable bioenergy transitions in low- and middle-income countries (Koubaa, 2024). Importantly, the PM-CPH co-digestion model also suggests a structurally complementary partnership between poultry farmers and cocoa producers, in which nitrogen-rich manure and carbon-rich cocoa residues are jointly valorized through shared anaerobic digestion infrastructure. Within such a cooperative framework, the resulting digestate could be redistributed to cocoa farms as a low-cost organic soil amendment, thereby closing local nutrient loops, improving soil organic matter recovery, and reducing dependence on mineral fertilizers. This reciprocal integration between livestock and perennial crop systems strengthens the socioeconomic attractiveness of the process while reinforcing circular resource flows at the territorial scale.

Overall, the results demonstrate that PM-CPH co-digestion extends beyond laboratory-scale biogas optimization and constitutes a potentially deployable low-input bioenergy strategy for decentralized agricultural regions. By coupling renewable energy recovery with nutrient recycling, waste mitigation, and climate-smart agricultural practices, the proposed system aligns with multiple Sustainable Development Goals related to affordable clean energy, responsible production, climate action, and sustainable agriculture (Figure 10). These findings therefore provide scientifically grounded evidence relevant to policy formulation, rural bioenergy planning, and future scale-up of anaerobic digestion systems in sub-Saharan Africa and comparable agroecological contexts.

## 4 Conclusion

This study demonstrates that anaerobic co-digestion of PM and CPH offers a technically robust, synergistic pathway to enhance biogas production from regionally abundant agricultural residues. Standardized biogas potential assessment tests (also known as biochemical methane potential assay) confirmed that balancing nitrogen-rich and carbon-rich substrates significantly improves process performance compared to mono-digestion, mitigating ammonia-related inhibition in PM and structural recalcitrance in CPH. The optimal configuration (C/N 25 under mesophilic conditions) achieved 348.65 ± 10.44 mL/g VS, corresponding to increases of 65% and 42% relative to PM and CPH mono-digestion, respectively. Process performance was primarily governed by substrate stoichiometry rather than thermal intensification. Maintaining a C/N ratio between 25 and 30 under mesophilic conditions (37 °C) ensured stable, reproducible yields, whereas under thermophilic conditions, the additional benefit was limited unless carbon-rich fractions dominated the mixture. These findings identify nutrient balance as the principal control parameter for process optimization in PM-CPH systems. Importantly, enhanced biogas production was achieved without chemical pretreatment, external buffering, or energy-intensive interventions, underscoring the intrinsic stabilizing synergy between the two substrates. From a technological readiness perspective, the results establish a validated laboratory-scale foundation and indicate a realistic pathway toward pilot-scale continuous operation through substrate management rather than technological complexity. Overall, PM-CPH co-digestion is a scalable, low-input strategy for decentralized waste-to-energy systems, contributing to the valorization of agricultural residues and the development of the circular bioeconomy. Future research should prioritize long-term continuous operation, pilot-scale validation, and integrated assessment of digestate management to support practical deployment in agro-industrial contexts.

## Data Availability

The raw data supporting the conclusions of this article will be made available by the authors, without undue reservation.
